# Atopic dermatitis in women: special considerations in the childbearing years

**DOI:** 10.1097/JW9.0000000000000151

**Published:** 2024-06-10

**Authors:** Rodolfo Valentini, Mona Shahriari

**Affiliations:** a University of Connecticut School of Medicine, Farmington, Connecticut; b Department of Dermatology, Yale University, New Haven, Connecticut; c Central Connecticut Dermatology, Cromwell, Connecticut

**Keywords:** atopic dermatitis, inflammation, lactation, pregnancy, treatment, women

## Abstract

**Background:**

Atopic dermatitis (AD) is one of the most common inflammatory dermatoses in adults. Women are disproportionately impacted by AD and report significant impacts on quality of life compared to men.

**Objective:**

Given the absence of formal guidelines for the treatment of AD in women of childbearing age, we will review special considerations for treating women of childbearing age with AD to ensure consistent care and optimal outcomes for these patients.

**Methods:**

PubMed and Google Scholar databases were searched for relevant articles from database inception through May of 2023.

**Results:**

There are several treatments including topical therapies, systemic therapies, and phototherapy that are considered safe during preconception, pregnancy and breastfeeding. Given the negative consequences of uncontrolled AD for both the mother and the unborn baby, the risks and benefits of potential therapies should be reviewed with all women of childbearing age suffering from AD.

**Limitations:**

The gold standard in recommending therapies is randomized controlled trials; however, pregnant and lactating women are often excluded from these trials.

**Conclusion:**

Through shared decision-making between the dermatologist, obstetrician, and patient, the risks and benefits of any therapy should be thoroughly discussed and considered with all women of childbearing age, to optimize care and outcomes for this unique population.

**What is known about this subject regarding women and their families?**

- Women, in particular women of childbearing age, are disproportionately impacted by atopic dermatitis (AD).
- Moreover, studies suggest menstrual or premenstruation worsening of AD in some women.
- During pregnancy, there is a shift towards a T-helper 2 predominant state, which can lead to deterioration and worsening of AD.
- However, due to concerns about the perceived safety of topical and systemic therapies during pregnancy, many women discontinue their AD therapies during pregnancy.
- Though the risks and benefits of every AD therapy should be carefully considered for women of childbearing age, the consequences of uncontrolled AD should also be considered when determining appropriate therapy.

**What is new from this article as messages for women and their families?**

- This article includes recommendations for the use of our newly food and drug administration-approved therapies for AD, including injectable biologics and oral small molecules, during preconception, pregnancy, and breastfeeding.

## Introduction

Atopic dermatitis (AD) is a chronic, relapsing, inflammatory skin disease with a heterogeneous clinical presentation.^1^ Though, historically, it has been considered a disease of childhood, AD remains one of the most common inflammatory dermatoses in adults, second only to acne.^2,3^

Males and females are not equally affected by AD.^2^ Studies show that AD is more common in males before the age of 11.^4^ However, with puberty, there is a shift towards AD impacting more women than men with this female predominance continuing into adulthood and impacting women of childbearing age.^2–4^ It is postulated that female sex hormones may modulate the course of AD through immune dysregulation and skin barrier dysfunction. This is evidenced by data showing menstrual/premenstruation and pregnancy-related deteriorations in the signs and symptoms of AD in about 50% of women with AD.^5^ Moreover, studies reveal that AD, in particular moderate to severe disease, impairs the quality of life of women more than men, and can even contribute to infertility. This underscores the importance of adequate disease control, especially in women.^3,4^

In this article, we will review the special considerations for treating women with AD, especially those of reproductive age, and the safety of our current therapeutics for this population to ensure optimal outcomes.

## Pathophysiology of atopic dermatitis

The pathogenesis of AD is multifactorial and involves a complex interplay between genetic, immune, and environmental risk factors. Genetic aberrations in structural proteins like ceramides and filaggrin can cause initial barrier dysfunction; this in combination with microbiome dysbiosis at the surface of the skin contributes to allergens and microbes penetrating the skin and activating key pro-inflammatory cytokines of the T-helper 2 (Th2) axis.^6–9^ These pro-inflammatory cytokines result in a positive feedback loop that leads to keratinocyte disruption, an amplification of barrier dysfunction, and further inflammation, which contributes to the signs and symptoms of AD.

## Atopic dermatitis and pregnancy

Classic AD is the most common general skin disease in pregnancy accounting for one-third to half of all gravid dermatologic ailments.^10,11^ Clinically and histologically, the AD seen in pregnant women is similar to the AD in nonpregnant women. It has been reported that 60 to 80% of patients will develop eczema for the first time when pregnant, most often by the end of the second trimester. Moreover, many women with a history of AD will experience worsening of their disease during this same time frame.^10,12^

The deterioration of AD during pregnancy is multifactorial. During pregnancy, there is a down-regulation of the Th1 pathway, which is involved in cellular immune responses, and a shift towards a Th2 response. This is to reduce immunologic responses against the fetus and ensure the continuation of the pregnancy.^13,14^ It is thought that this shift towards a Th2 response is secondary to the impact of high concentrations of estrogen and progesterone on the Th2 pathway and the skin barrier.^5^

Atopic eruption of pregnancy (AEP) is the most common pregnancy-specific dermatosis and is oftentimes mistaken for classic AD. AEP is thought to be due to immunological changes during pregnancy that lead to a Th2-predominant state.^15^ AEP used to be called eczema of pregnancy due to its histopathologic and clinical similarity to classic AD and incidence in patients with a history of AD.^11,13^ However, AEP is a distinct eczematous disease with mean onset at week 18 of pregnancy, majority of cases presenting before the third trimester, and most patients reporting no prior history of AD.^6,15^ Proper work-up of AEP includes excluding intrahepatic cholestasis of pregnancy, which is also part of the differential diagnosis for pruritus in pregnancy and should be considered if serum bile acids are elevated.^15^

The classic clinical presentation of AD during pregnancy can aid in timely diagnosis and treatment. Still, effective and safe treatment during pregnancy can be challenging. One recent study looked at trends in AD medication use 180 days before pregnancy and during all 3 trimesters. They found that systemic medications were stopped, by at least half of patients, by the first trimester. Topical corticosteroid use also dropped, going from 55.4% of the total cohort 180 days before pregnancy to 5.9% by the first trimester.^8^ Interestingly, systemic steroids saw the least decline of all systemic therapies and some patients even initiated treatment during pregnancy. A major limitation of this study was that the reason for treatment cessation was not captured. So, it is unclear whether the cessation of therapy was due to the resolution of the AD or concerns about the safety of these medications during pregnancy.

These findings may be a result of provider discomfort and hesitancy to prescribe systemic during pregnancy, especially since this trend has been seen across multiple systemic diseases.^9–11^ It may also be a result of patient hesitancy to be on a systemic medication while pregnant, with one study finding that 37% of pregnant patients stopped taking a regularly prescribed medication due to concerns about the risks of that therapy.^11^

Though topical and systemic treatment of AD during pregnancy can impact the unborn baby, inadequately controlled AD, in and of itself, can have negative consequences for both the mother and the unborn baby. Poor disease control results in microbiome dysbiosis and an increase in *Staphylococcus aureus* colonization at the surface of the skin which can result in maternal Staphylococcal infections.^12^ Staphylococcal infections are always concerning, but can be dangerous in pregnancy due to their association with premature rupture of membranes and neonatal septicemia.^13^ Along the same lines, eczema herpeticum is more common with poorly controlled AD, and can be particularly concerning during pregnancy as disseminated infection can cause maternal and fetal demise.^16^ Finally, uncontrolled AD can result in a systemic pro-inflammatory state that has been shown to be associated with decreased brain development and prematurity.^14^

## Therapeutics for atopic dermatitis

In all women of childbearing age with AD, family planning should be discussed and reviewed on a regular basis. In those women who are sexually active but not utilizing any form of birth control, treatment selection should keep the notion of an unplanned pregnancy in mind. Moreover, all patients should be reminded to inform their provider if they become pregnant.

Initial management of AD includes disease education, eliminating triggers, promptly treating skin infections, and aggressive skincare regimens. Proper skin care with hydration using thick, bland emollient creams is central to any AD treatment regimen.^17–19^ Topical antipruritic medications including diphenhydramine and chlorpheniraine play a role in breaking the itch-scratch cycle and are unlikely to be harmful to a fetus due to lack of systemic absorption.^7,8^ Bleach baths—including sodium hypochlorite and potassium permanganate—are an adjunct in the management of AD.^17,20^ No study has been performed on bleach exposure in pregnancy, but its historic and continued use in commercial pools without identifiable fetal consequences is reassuring. The following sections discuss considerations for the use of routine AD medications in pregnancy. These considerations are summarized in Table 1.

**Table 1:** Summary of therapeutic recommendations for AD in pregnancy and lactation

Topical therapy	Relevant information
Topical corticosteroids	• Use lowest potency possible • Potent and very potent should not exceed 300g over the gestational course to prevent low birth weight
Topical calcineurin inhibitors	• Aids in limiting use of topical corticosteroids • May use during pregnancy; no human data available though risk of fetal harm is not expected due to minimal systemic absorption
Crisaborole	• No data available • Not recommended in pregnancy or breastfeeding
Ruxolitinib	• No data available • Not recommended in pregnancy or breastfeeding
Phototherapy	• Narrow band UV-B safe for pregnancy and breastfeeding but may induce or worsen melasma. Consider folate supplementation in patients in particular in Fitzpatrick skin types I–III • Psoralen and UV-A therapy contraindicated due to psoralen teratogenicity
Systemic therapy	
Systemic corticosteroids	• Should only be used as rescue therapy during pregnancy for no longer than 2–3 weeks. • European guidelines suggest a max of 0.5 mg/kg/day. • Repeated rounds may cause growth retardation in fetus • Safe during breastfeeding
Cyclosporine	• Can be used for AD that is refractory to other therapies and where benefits to mother outweigh risks to fetus • Increases risk of low birth weight and maternal hypertension • Maternal renal function and blood pressure should be monitored • Not recommended during breastfeeding
Azathioprine	• Should not be given in pregnancy without carefully weighing the risks and benefits, and should be avoided when possible since there are better alternatives • Risks include higher rates of spontaneous abortion, increased prematurity, increased preterm delivery, neonatal leukopenia, and neonatal pancytopenia • Weigh risks and benefits during breastfeeding
Dupilumab	• No identified adverse maternal or fetal outcomes, but limited data in pregnancy, so proceed with caution • No current data in breastfeeding • Observational cohort study looking at the incidence of adverse pregnancy outcomes is underway
Tralokinumab	• Limited data on use in pregnancy, so proceed with caution • No current data in breastfeeding
JAK inhibitors: - Upadacitinib - Abrocitinib	• No human data in pregnancy; animal models show potential teratogenicity • Contraindicated in pregnancy • Wait 4 weeks following last dose to attempt conception • Avoid breastfeeding during, and 6 days following last dose
Methotrexate	• Contraindicated in pregnancy and breastfeeding • Can cause skeletal abnormalities and embryopathy • Breastfeeding can cause immunosuppression in the infant
Mycophenolate mofetil	• Causes fetal CNS, kidney, and cardiovascular defects • High miscarriage rates • Contraindicated in pregnancy and breastfeeding • Should be stopped 3 months before conception and not restarted until completion of breastfeeding • Part of the REMS program

AD, atopic dermatitis; REMS, risk evaluation and mitigation strategy.

## Topical therapies

### Topical corticosteroids

When skin barrier repair through skincare regimens is not enough, most clinicians initiate therapy with low to high potency (class 2–6) topical corticosteroids (TCS). Superpotent (class 1) steroids can be used judiciously for short periods of time due to concerns about local and systemic side effects with long-term use.^17^ Pregnancy recommendations differ in that potent TCS should be used sparingly and superpotent TCS avoided completely.^18,21^ Potent and very potent TCS are associated with lower birth weights when the total gestational dosage exceeds 300g.^9^ This is especially true when used on large surface areas, since percutaneous absorption of the TCS can result in unwanted systemic adverse events. TCS is not of significant concern in breastfeeding, although application to the nipple should be avoided when possible.^7,8,10^

### Topical calcineurin inhibitors

Topical calcineurin inhibitors (TCIs) are commonly used to limit the local and systemic side effects of long-term steroid use and for their safety in sensitive areas such as the face or intertriginous regions.^17,18^ Clinical trials looking specifically at the safety of TCIs in pregnancy have not been conducted. However, previous reports indicate a limited systemic absorption of TCIs through the skin barrier when standard doses for AD are used.^22^ Moreover, food and drug administration (FDA) guidance states that TCIs may be used during pregnancy; no human data is available, though the risk of fetal harm is not expected based on minimal systemic absorption.^23^ TCIs do carry boxed warnings for possible increased risk of lymphoma and skin cancers, and as such, shared decision-making with expectant mothers is key to ensure they are aware of the benefits and potential risks of therapy. It is thought that TCIs are not excreted into breast milk, although it is not recommended for use on the nipple when breastfeeding.^21^

### Crisaborole

Crisaborole is a phosphodiesterase 4 inhibitor with no data on use during the preconception period, pregnancy, or breastfeeding. It is therefore not recommended for use in pregnancy or breastfeeding until reproducible and reliable evidence can elucidate its safety.^16^

### Ruxolitinib

Ruxolitinib is a topical Janus kinase inhibitor (JAK-I) with no current data on its safety during the preconception period, pregnancy, or breastfeeding. Therefore, it is currently not recommended for use in these groups.^24^

## Phototherapy

Narrow Band UV-B (NBUVB) treats AD by reducing inflammation in the skin.^25,26^ Though clinical trials looking at the safety of NBUVB in pregnant women have not been conducted, pregnant women have been exposed to sunlight as a result of outdoor jobs without any known harm to the fetus.^27^ A well-known risk of chronic UV exposure is an increased risk of skin cancer, which should be considered in anyone who is at increased risk of skin cancer at baseline.^25,26^ When specifically looking at risks in pregnant women, there is an increased risk of new onset or worsening melasma.^28,29^ Though the latter is not harmful to the mother or the unborn child, it can have significant psychosocial impacts on the mother, as such, the use of facial shielding is recommended.^7^

Given the minimal risks, NBUVB is considered safe for use during preconception, pregnancy, and breastfeeding. Folic acid supplementation is recommended in women of childbearing age receiving NBUVB phototherapy due to reductions in serum folate levels with long-term treatment.^30^ Psoralen and UV-A therapy is contraindicated in pregnancy due to the mutagenic and teratogenic properties of psoralen.^31^ Psoralen is also contraindicated during breastfeeding due to its excretion into breast milk.^32^

## Systemic therapies

Systemic therapy options are limited for pregnant and breastfeeding women due to concerns about the adverse effects on the mother and her unborn child. So, the risks/benefits of every medication should be weighed before commencing therapy.

### Systemic corticosteroid therapy

Systemic corticosteroid therapy for AD is only recommended as rescue therapy during pregnancy and is not advised for longer than 2 to 3 weeks.^28,29^ Moreover, repeated rounds of steroids have been associated with decreased birthweight, and growth retardation, irrespective of the gestational age of the fetus at the time of exposure.^21^ Some European guidelines suggest a max of 0.5 mg/kg/day for a maximum of 2–3 weeks, preferring steroids with high inactivation rates to help limit or even prevent fetal systemic corticosteroid therapy exposure.^28^ With respect to breastfeeding, no adverse effects have been reported in breastfed infants as excretion into breast milk is low, with breast milk concentrations 0.1% or less of the ingested dose.^7^

### Cyclosporin

Cyclosporin (CSA) is an oral calcineurin inhibitor. Most data on the safety of CSA use in pregnancy comes from solid organ transplant patients.^11–14^ Though CSA crosses the placenta, all studies show that at doses of less than 8–10 mg/kg/day, it does not have teratogenic properties. However, an increased risk of intrauterine growth restriction, prematurity and maternal hypertension and pre-ecplampsia may exist.^11–14^ As such, CSA can be considered judiciously in severe AD cases that are recalcitrant to other therapies, and the benefit to the mother justifies the potential risks to the fetus. A risk-benefit discussion is important before initiation, especially during the first trimester, and maternal renal function and blood pressure should be monitored during therapy.^10,11^ Cases of breastfeeding mothers on CSA for solid organ transplant have reported no toxicities in their children; however, using CSA for AD while breastfeeding is currently not recommended.^11^

### Azathioprine

Azathioprine (AZA) is a purine analogue that blocks purine synthesis. It is anti-inflammatory at doses used for AD, but cytotoxic at higher dosages.^17^ Though we do not have consistent reports of congenital defects caused by treatment with AZA in patients with other inflammatory conditions,^7,11^ using AZA in pregnancy is controversial due to risks of neonatal leukopenia and pancytopenia, higher rates of spontaneous abortion, increased prematurity, and increased preterm delivery.^7,18^ The FDA label states that AZA should not be given during pregnancy without carefully weighing risks and benefits and wherever possible, its use should be avoided. The European task force on atopic dermatitis has also recommended the avoidance of AZA during pregnancy as there are better alternatives.^18,19^ So, the use of AZA should be minimalized or avoided where possible, with obstetric consultation for appropriate monitoring when prescribed.^10^ With respect to lactation, weigh the risks and benefits while breastfeeding, but human data has shown a low risk of infant harm.^18,19^

### Dupilumab

Dupilumab is a monoclonal antibody targeting the IL-4 receptor alpha thereby blocking the signaling of IL-4 and IL-13 which are key cytokines in the development of AD.^33^ It is a targeted biologic that is currently FDA-approved for the treatment of patients with refractory moderate to severe AD down to 6 months of age.^20^

Though the favorable safety profile of this drug has been a welcome addition to our systemic therapy toolbox, there is still limited data on the safety of dupilumab in pregnancy and breastfeeding. Available data from case reports and case series with dupilumab use in pregnant women have not identified a drug-associated risk of major birth defects, miscarriage, or adverse maternal or fetal outcomes.^16,21–23,25–28,32,34^ However, human IgG antibodies are known to cross the placental barrier; therefore, dupilumab may be transmitted from the mother to the developing fetus. Currently, little is known about the impacts of this in utero exposure on the developing fetus and the newborn baby. Moreover, there is no data on the effects of dupilumab on milk production or breastfed infants.

It should be noted that 2 international respiratory medical groups believe that dupilumab may be safe throughout pregnancy and breastfeeding based on data from omalizumab, but further studies are necessary.^24,35–37^ Currently, an observational retrospective cohort study is underway looking at the incidence of adverse pregnancy outcomes in women with AD who are treated with dupilumab during pregnancy in comparison to women with AD who were not exposed to dupilumab during pregnancy. This study is set to conclude in 2027 and will be valuable in understanding the safety of this drug in pregnant or breastfeeding women.^29^ Until this data becomes available, the decision for perinatal use should be a shared decision that involves input from the patient, dermatologist, obstetrician, and physicians treating other type 2 inflammatory diseases.

### Tralokinumab

Tralokinumab is a monoclonal antibody that binds the IL-13 cytokine thereby inhibiting is interaction with its receptor.^30^ Approved in December 2021, there is limited data on the use of tralokinumab during pregnancy, and no data on the safety of tralokinumab while breastfeeding.^31,38^ As such, perinatal use should include a shared decision-making conversation that reviews the risks and benefits of this therapy.

## Contraindicated medications

Here we will briefly review the medications are contraindicated in women who are pregnant or breastfeeding.

### JAK inhibitors

The JAK-Is are a new class of systemic drugs for AD and include upadacitinib (Rinvoq), and abrocitinib (Cibinqo).^39^ They are targeted and highly efficacious with a more favorable safety profile than our traditional systemics like CSA, methotrexate, or AZA. While there is no data to support their use during pregnancy, preclinical animal studies have shown potential teratogenicity with JAK-Is, as such, they are contraindicated in pregnancy.^39,40^ The most recent guidelines suggest waiting at least 4 weeks following the last dose of any JAK-I to attempt conception. Data on the presence of JAK-Is in human milk, the effects on the breastfed infant, or the effects on milk production are not known. As such, the recommendation is to avoid breastfeeding while taking a JAK-I and for 6 days after the last dose.^41^

### Methotrexate

Methotrexate use in pregnancy can result in significant skeletal abnormalities and embryopathy. And even though less than 10% is excreted in breast milk, it can still result in immunosuppression in an infant that is exposed through breastfeeding.^9^ As such, it is contraindicated in pregnancy and breastfeeding. Suggested preconception cessation varies between 1 and 6 months when possible.^9,42,43^

### Mycophenolate mofetil

Mycophenolate mofetil (MMF) use in pregnancy can result in central nervous system, kidney, and cardiovascular defects in the fetus, as well as high miscarriage rates; it is for these reasons that MMF is part of the risk evaluation and mitigation strategy drug safety program.^9^ Given that it has the lowest strength of recommendation of all systemic therapies for AD due to its variable efficacy, the benefits certainly do not outweigh the risks and it is contraindicated in pregnancy. It is also contraindicated in breastfeeding due to insufficient safety data on the impacts of MMF on milk production and the breastfed infant. MMF should be stopped 3 months before pregnancy until birth or cessation of breastfeeding—whichever comes last.^43,44^

## Conclusion

AD is one of the most common inflammatory dermatoses of adulthood with a particularly high quality of life burden on women. The impact of female sex hormones on the immune pathways that govern AD results in the deterioration of AD during the premenstrual period and during pregnancy, which makes women, especially those of childbearing age, at increased risk of AD flares. However, safe and effective treatment of AD in this population can be challenging. Since pregnant women are excluded from clinical trials, formal guidelines for the treatment of AD during preconception, pregnancy, and breastfeeding are lacking. However, the negative consequences of uncontrolled AD for both the mother and unborn baby cannot be overstated. Through shared decision-making between the dermatologist, obstetrician, and patient, the risks and benefits of any therapy should be thoroughly discussed and considered with all women of childbearing age, to optimize care and outcomes for this unique population.

## Conflicts of interest

The authors made the following disclosures: R.V.: Consultant: McGraw Hill Education. M.S.: Consultant (honoraria): AbbVie, Arcutis, Bristol Myers Squibb, Dermavant, Janssen, Leo Pharma, Lilly USA, Novartis, Novan, Galderma, Ortho Dermatologics, Sanofi-Genzyme, Regeneron, UCB. Speaker: Abbvie, Arcutis, Bristol Myers Squibb, Lilly USA, Janssen, Dermavant, Leo Pharma, Sanofi-Genzyme, Regeneron, UCB. Investigator: AbbVie, CorEvitas Psoriasis Registry, CorEvitas Atopic Dermatitis Registry, Dermira, Lilly USA, Cara, Dermavant, Novartis, Union, Mindera.

## Funding

None.

## Study approval

N/A

## Author contributions

RV and MS: Contributed to the conception, design, and drafting of the manuscript. They both critically reviewed the final version of the manuscript and take full responsibility for the accuracy of the work.
